# Antipsychotic Dose Mediates the Association between Polypharmacy and Corrected QT Interval

**DOI:** 10.1371/journal.pone.0148212

**Published:** 2016-02-03

**Authors:** Corrado Barbui, Irene Bighelli, Giuseppe Carrà, Mariasole Castellazzi, Claudio Lucii, Giovanni Martinotti, Michela Nosè, Giovanni Ostuzzi, STAR NETWORK INVESTIGATORS

**Affiliations:** 1 WHO Collaborating Centre for Research and Training in Mental Health and Service Evaluation, Section of Psychiatry, University of Verona, Verona, Italy; 2 Division of Psychiatry, University College of London, UK, and Department of Medicine and Surgery, University of Milano Bicocca, Milan, Italy; 3 Department of Mental Health, Siena, Italy; 4 Department of Neuroscience, Imaging and Clinical Sciences, University of Chieti, Chieti, Italy; University of Verona, Ospedale Civile Maggiore, ITALY

## Abstract

Antipsychotic (AP) drugs have the potential to cause prolongation of the QT interval corrected for heart rate (QTc). As this risk is dose-dependent, it may be associated with the number of AP drugs concurrently prescribed, which is known to be associated with increased cumulative equivalent AP dosage. This study analysed whether AP dose mediates the relationship between polypharmacy and QTc interval. We used data from a cross-sectional survey that investigated the prevalence of QTc lengthening among people with psychiatric illnesses in Italy. AP polypharmacy was tested for evidence of association with AP dose and QTc interval using the Baron and Kenny mediational model. A total of 725 patients were included in this analysis. Of these, 186 (26%) were treated with two or more AP drugs (AP polypharmacy). The mean cumulative AP dose was significantly higher in those receiving AP polypharmacy (prescribed daily dose/defined daily dose = 2.93, standard deviation 1.31) than monotherapy (prescribed daily dose/defined daily dose = 0.82, standard deviation 0.77) (z = −12.62, p < 0.001). Similarly, the mean QTc interval was significantly longer in those receiving AP polypharmacy (mean = 420.86 milliseconds, standard deviation 27.16) than monotherapy (mean = 413.42 milliseconds, standard deviation 31.54) (z = −2.70, p = 0.006). The Baron and Kenny mediational analysis showed that, after adjustment for confounding variables, AP dose mediates the association between polypharmacy and QTc interval. The present study found that AP polypharmacy is associated with QTc interval, and this effect is mediated by AP dose. Given the high prevalence of AP polypharmacy in real-world clinical practice, clinicians should consider not only the myriad risk factors for QTc prolongation in their patients, but also that adding a second AP drug may further increase risk as compared with monotherapy.

## Introduction

Antipsychotic (AP) drugs as a group have long been known to have the potential to cause prolongation of the QT interval corrected for heart rate (QTc) [1;2]. Data on individual AP drugs are more controversial, with individual phenothiazines and butyrophenones (e.g., haloperidol) carrying a higher risk as compared with some individual second-generation AP drugs, such as quetiapine and olanzapine, which may have a moderate risk, or aripiprazole, possibly showing a lower potential to cause QTc prolongation [3;4]. However, as many other risk factors for QTc prolongation have been identified [2;5], these data remain difficult to interpret and somehow controversial [6].

As the risk is considered dose-dependent [3;7;8], it may also be associated with the number of AP drugs concurrently prescribed, as AP polypharmacy has consistently been shown to be associated with increased combined equivalent AP dosage [9;10]. However, a recent systematic review of 10 clinical trials, 4 observational studies, and 7 case reports, failed to find that AP polypharmacy worsens QTc interval, although it pointed out that the evidence is scarce and inconsistent [11]. In a cross-sectional survey carried out to estimate the prevalence of QTc lengthening in a sample of people with psychiatric illnesses, we showed that AP polypharmacy was positively associated with QTc prolongation, but the sample of included patients was quite heterogeneous in terms of cardiovascular disorders and use of medications [4]. Besides, whether AP dose mediates the association between polypharmacy and QTc interval has not been investigated yet, largely remaining unknown.

The present study analysed the mediational role of AP dose in the relationship between polypharmacy and QTc interval. We additionally investigated whether haloperidol and individual second-generation AP drugs are associated with QTc prolongation, taking into account AP polypharmacy and dose.

## Materials and Methods

### Study Participants

This study is based on data from a cross-sectional survey that investigated the prevalence of QTc lengthening among people with psychiatric illnesses in Italy [4]. The study was carried out in 35 Italian psychiatric services that are part of the STAR (*Servizi Territoriali Associati per la Ricerca*) Network, a research group established to produce scientific knowledge by collecting data under ordinary clinical practice. The study design has already been presented elsewhere [4]. Briefly, during a three-month recruitment period, a consecutive unselected series of both in- and out- patients were invited to participate. Inpatients aged 18 or above were included if they gave informed written consent, performed an ECG during hospital stay, and were receiving pharmacological treatment with psychotropic drugs on the day of ECG recording. For inpatients with more than one ECG during hospital stay, the first was considered. Outpatients aged 18 or above were included if they gave informed written consent, underwent ECG examination during the recruitment period, and were receiving pharmacological treatment with psychotropic drugs on the day of ECG performance. For outpatients with more than one ECG during the recruitment period, we considered the earliest. A specific psychiatric diagnosis was not a requirement for inclusion in the study. The study received ethical approval by the Ethics Committee of the *Azienda Ospedaliera Universitaria Integrata*, Verona (Approval Number 2409) and by the Ethics Committee of each participating site, and all participants gave their informed written consent. As all participants had full mental capacity, no specific measure was adopted to determine capacity to consent.

### Data Collection

Socio-demographic and clinical characteristics were collected from medical records, including ICD-10 psychiatric diagnosis, alcohol/substances use, recruitment setting, being admitted for drug overdose, electrolyte (sodium, photassium, calcium, chloride) imbalances, cardiovascular disorders, drug treatments for psychiatric disorders, drug treatments for other disorders and antipsychotic prescribed daily doses. Drugs were classified following the Anatomical Therapeutic Chemical Classification (ATC) system and, following the Arizona Cert (AzCERT), those that can cause QTc lengthening under normal clinical usage were identified.

Antipsychotic drug doses (N05A of the ATC group excluding N05AN, lithium) were converted into multiples of the Defined Daily Dose (DDD) for each drug by dividing the prescribed daily dose (PDD) by the DDD [PDD/DDD] [12]. The DDD is the international unit of drug utilisation approved by the World Health Organisation for drug use studies. It is a theoretical unit of measurement defined as the assumed average maintenance daily dose for a drug, used for its main indication in adults. Expression of drug use in terms of multiples of DDDs allows calculating, for each patient, a cumulative measure of drug consumption taking into account the concurrent use of more than one agent. A PDD/DDD ratio of one indicates that the dose prescribed is equal to the DDD of that drug; a ratio greater than one indicates a dosage higher than the DDD of that drug, while a ratio lower than one means a dose lower than the DDD of that drug [13].

The QTc interval estimation was obtained in each participating site from standard 12-lead ECG. The most common way for interpreting the QT interval is to divide its value by the square root of the RR interval expressed in seconds, namely, by using Bazett's formula for correction. The QTc was determined by examining lead II with automatic data acquisition and was confirmed by a cardiologist who was blind to the patient’s clinical condition.

### Sample Definition

**Fig 1:**
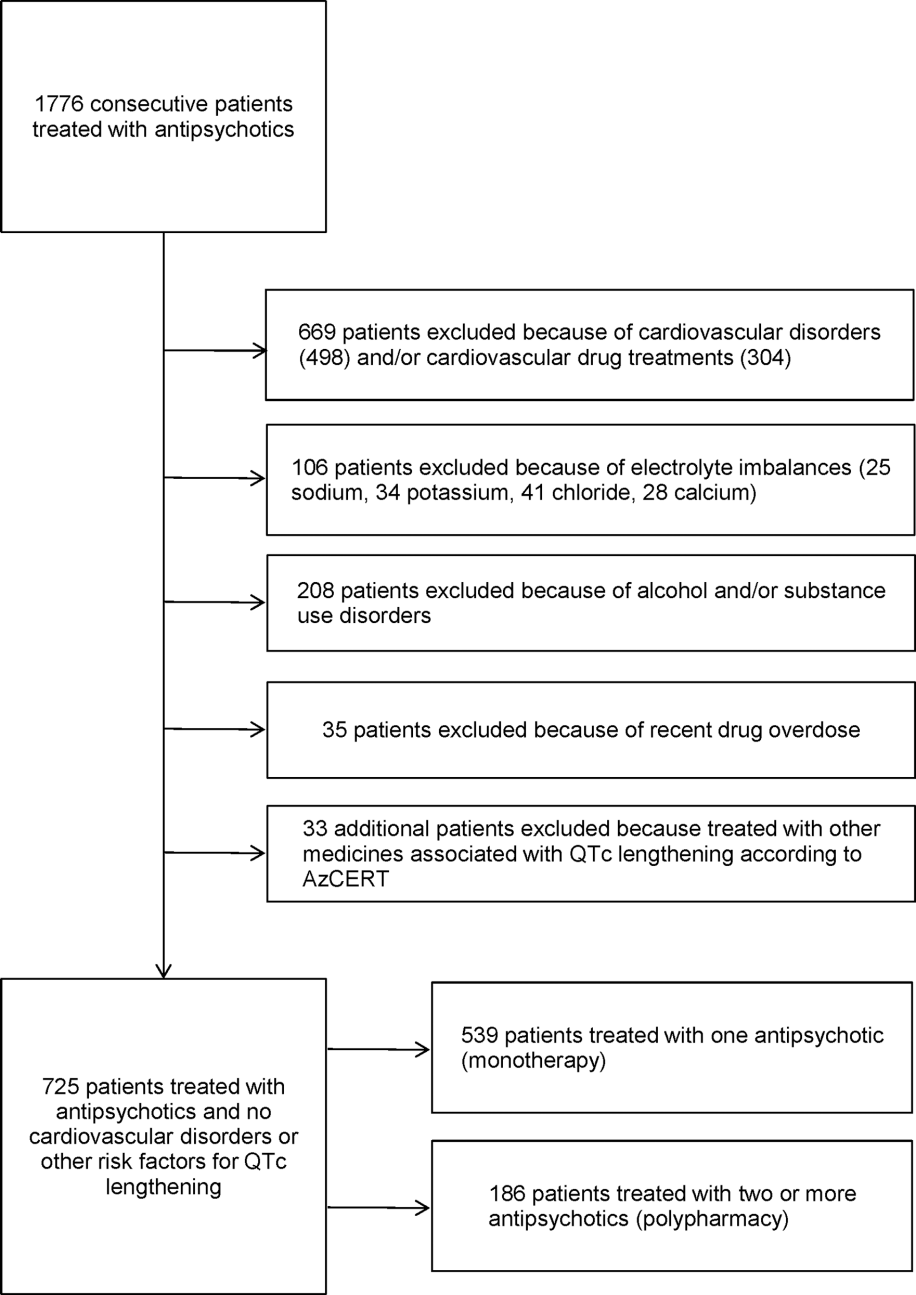
Flow chart of inclusion and exclusion criteria of cohort of patients treated with antipsychotic drugs.

For the purposes of this analysis, from the entire sample of recruited patients we excluded those with risk factors for QTc lengthening (Fig 1). Out of 1776 consecutive patients treated with AP drugs, we excluded 1051 patients for the following reasons: cardiovascular disorders and/or cardiovascular drug treatments; electrolyte imbalances when the ECG was performed; alcohol and/or substance use disorders; drug overdose as reason for admission; use of any other medicines associated with QTc lengthening according to AzCERT (Fig 1).

### Data Analysis

We first tested AP polypharmacy (use of two or more AP drugs) as a dichotomous variable for evidence of association with socio-demographic information, clinical data and drug use. Chi-squared statistics were calculated for pairs of dichotomous variables, and Mann-Whitney statistics were used to analyse continuous variables by AP polypharmacy.

Subsequently, AP polypharmacy was tested for evidence of association with AP dose and QTc interval in univariate nonparametric test for trend across ordered groups, which is an extension of the Wilcoxon rank-sum test [14]. As additional step, the Baron and Kenny mediational model was applied [15;16]. According to this approach, a mediating role of a variable exists when four conditions are met: (i) the predictor variable (AP polypharmacy) must be significantly related to the outcome variable (QTc interval); (ii) the hypothesized mediator (AP dose) must be significantly related to the predictor variable (AP polypharmacy); (iii) the mediator (AP dose) must be significantly related to the outcome (QTc interval); and (iv) the relationship between the predictor (AP polypharmacy) and the outcome (QTc interval) must be attenuated when controlling for the mediator (AP dose). If the predictor remains significant when the mediator is controlled for, mediation is deemed to be partial. When controlling for the mediator renders the predictor non-significant, mediation is deemed complete [15;16]. The four conditions of the Baron and Kenny mediational model were tested by means of four linear regression analyses, adjusting for the following confounding variables: sex (female = 1, male = 0), age (years, continuous variable), psychosis or related disorder (no = 0, yes = 1), length of illness (years, continuous variable), inpatient status (no = 0, yes = 1), treatment with AD drugs (no = 0, yes = 1), treatment with mood stabilisers (no = 0, yes = 1). A nonparametric bootstrap method of statistical accuracy was used, assuming that the observed distribution of the present sample was a good estimate of the true population distribution [17].

In addition, we tested whether use of haloperidol, risperidone, olanzapine, clozapine, quetiapine, paliperidone or aripiprazole was associated with QTc interval. For each of these drugs, nonparametric bootstrap linear regression analyses were conducted including (i) the confounding variables reported above (first analysis), (ii) the confounding variables reported above plus AP polypharmacy (second analysis), and (iii) the confounding variables reported above plus AP polypharmacy and AP dose (third analysis). Statistical analysis was carried out with STATA 13.

## Results

### Patient Characteristics

A total of 725 patients were included in this analysis (Fig 1). The mean QTc interval was 412.40 ms in men (SD 28.35) and 417.70 ms (SD 32.20) in women. In men, 30 patients (9.23%) showed a QTc longer than 450 ms, while in women 12 patients (3%) showed a QTc longer than 470 ms. Of the whole sample, 186 (26%) were treated with two or more AP drugs (AP polypharmacy). The main socio-demographic and clinical characteristics are presented in Table 1. Patients receiving AP polypharmacy were more often men, with psychotic disorders, and slightly younger than patients treated with one AP only. In terms of drug treatments, use of haloperidol, clozapine, quetiapine and aripiprazole was more frequent in patients treated with AP polypharmacy (Table 1). AD drugs were more often used in those receiving one AP only.

**Table 1:** Demographic and clinical features of patients treated with one (monotherapy) or two or more (polypharmacy) antipsychotic (AP) drugs.

		AP monotherapy (539)		AP polypharmacy (186)		
		N	%	N	%	P-value
Sex	men	230	42.67	95	51.08	0.047
	women	309	57.33	91	48.92	
Age (years)	18–30	95	17.63	27	14.52	0.003
	31–50	231	42.86	109	58.60	
	51–70	183	33.95	42	22.58	
	71–93	30	5.57	8	4.30	
Psychosis	no	293	54.36	76	40.86	0.001
	yes	246	45.64	110	59.14	
Length of illness (years)	< 1	53	10.86	11	7.59	0.061
	1–5	145	29.71	29	20.00	
	6–10	79	16.19	28	19.31	
	11–20	109	22.34	35	24.14	
	> 20	102	20.90	42	28.97	
Inpatients	no	173	32.10	47	25.27	0.081
	yes	366	67.90	139	74.73	
***Haloperidol***	no	454	84.23	105	56.45	0.001
	yes	85	15.77	81	43.55	
***Risperidone***	no	480	89.05	164	88.17	0.742
	yes	59	10.95	22	11.83	
***Olanzapine***	no	430	79.78	147	79.03	0.828
	yes	109	20.22	39	20.97	
***Clozapine***	no	500	92.76	156	83.87	0.001
	yes	39	7.24	30	16.13	
***Quetiapine***	no	445	82.56	131	70.43	0.001
	yes	94	17.44	55	29.57	
***Paliperidone***	no	522	96.85	178	95.70	0.460
	yes	17	3.15	8	4.30	
***Aripiprazole***	no	486	90.17	153	82.26	0.004
	yes	53	9.83	33	17.74	
***Antidepressant drugs***	no	346	64.19	142	76.34	0.002
	yes	193	35.81	44	23.66	
***Mood Stabilizers***	no	337	62.52	121	65.05	0.537
	yes	202	37.48	65	34.95	

### AP Polypharmacy, AP Dose and QTc Interval

**Fig 2:**
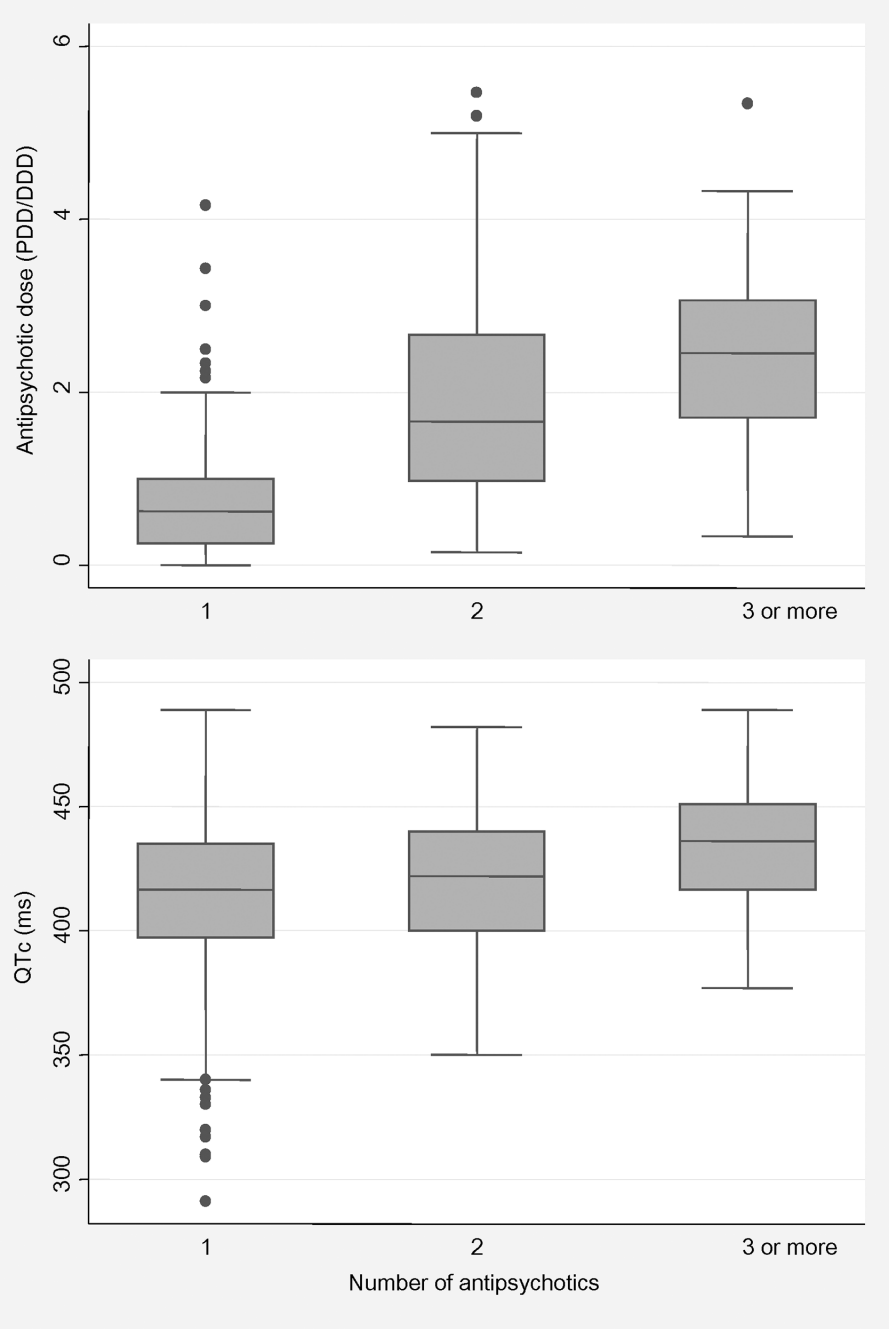
Distribution of antipsychotic dose and QTc interval by number of antipsychotics.

The mean AP dose was significantly higher in those receiving AP polypharmacy (PDD/DDD = 2.93, SD 1.31) than in those receiving AP monotherapy (PDD/DDD = 0.82, SD 0.77) (z = −12.62, p < 0.001). Similarly, the mean QTc interval was significantly longer in those receiving AP polypharmacy (mean = 420.86 ms, SD 27.16) than in those receiving AP monotherapy (mean = 413.42 ms, SD 31.54) (z = −2.70, p = 0.006). Fig 2 shows that the number of AP drugs received was significantly associated with AP dose (z test for trend = 12.35, p < 0.001) and QTc interval (z test for trend = 3.35, p = 0.001).

**Fig 3:**
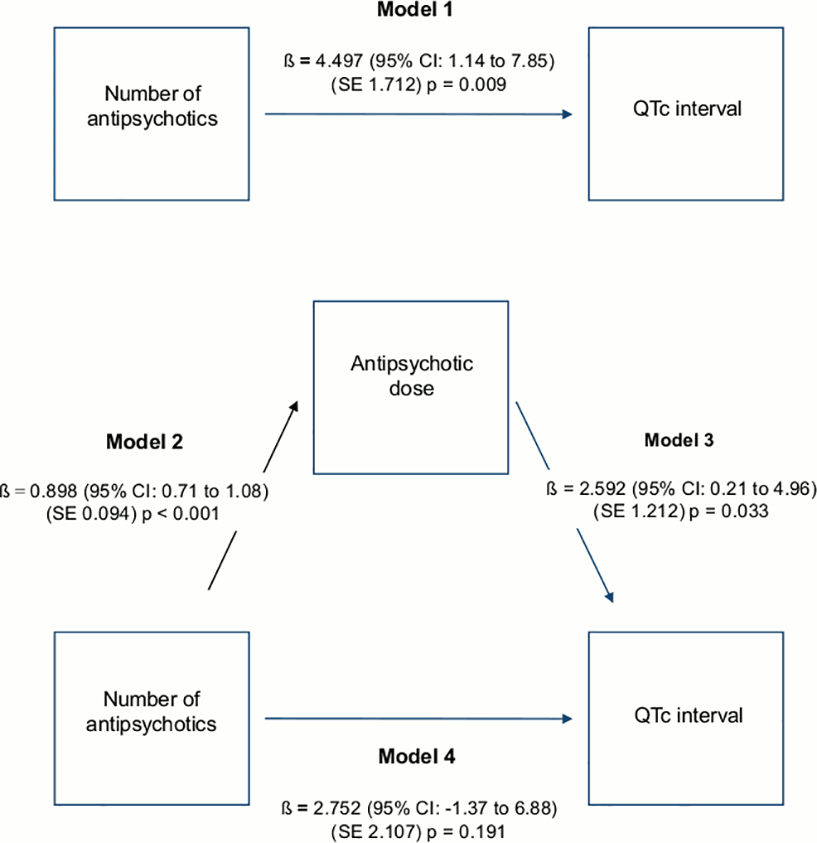
Mediational model showing both the direct and the mediated pathways of the relationship between antipsychotic polypharmacy and QTc interval. Observed coefficients (ß) with standard errors (SE) and p-values are reported.

Fig 3 presents the results of the Baron and Kenny approach. It shows that, after adjustment for confounding variables, AP polypharmacy was significantly associated with QTc interval (Model 1); AP polypharmacy was significantly associated with AP dose (Model 2); AP dose was significantly associated with QTc interval (Model 3); after controlling for AP dose, AP polypharmacy was no longer associated with QTc interval (Model 4). This suggests that AP dose mediates the association between polypharmacy and QTc interval.

### Individual AP Drug and QTc Interval

We tested whether haloperidol and individual second-generation AP drugs were associated with QTc lengthening (Table 2), controlling for AP polypharmacy and dose. We failed to find an association between individual AP drugs and QTc interval, but we observed that people on aripiprazole were significantly less likely to show QTc prolongation, which persisted after controlling for AP dose and polypharmacy (Table 2).

**Table 2:** Relationship between exposure to haloperidol and individual second-generation AP drugs and QTc interval.

Dependent variable	QTc								
Adjusted for	Sex, age, length of illness, diagnosis, setting, AD, MS			Sex, age, length of illness, diagnosis, setting, AD, MS, number of antipsychotics			Sex, age, length of illness, diagnosis, setting, AD, MS, number of antipsychotics and antipsychotic dose		
Drug	Coefficient (bias corrected 95% CI)	*z* value	*P* value	Coefficient (bias corrected 95% CI)	*z* value	*P* value	Coefficient (bias corrected 95% CI)	*z* value	*P* value
Haloperidol	3.64 (-2.85 to 10.13)	1.10	0.272	2.27 (-2.57 to 7.12)	0.92	0.358	2.82 (-2.10 to 7.76)	1.12	0.261
Risperidone	-2.87 (-10.84 to 5.09)	-0.71	0.479	-2.79 (-9.79 to 4.20)	-0.78	0.434	-2.08 (-9.74 to 5.57)	-0.53	0.593
Olanzapine	-0.84 (-5.00 to 3.31)	-0.40	0.690	-1.05 (-6.01 to 3.90)	-0.42	0.676	-1.86 (-7.15 to 3.42)	-0.69	0.490
Clozapine	-0.04 (-8.66 to 8.56)	-0.01	0.991	-0.88 (-11.50 to 9.74)	-0.16	0.871	-0.82 (-10.02 to 8.36)	-0.18	0.860
Quetiapine	4.57 (-1.51 to 10.66)	1.47	0.141	3.69 (-2.20 to 9.60)	1.23	0.222	3.92 (-2.34 to 10.20)	1.23	0.220
Paliperidone	-2.38 (-11.42 to 6.66)	-0.52	0.606	-2.47 (-11.91 to 6.97)	-0.51	0.608	0.46 (-8.95 to 8.03)	-0.11	0.915
Aripiprazole	-10.83 (-19.06 to -2.61)	-2.58	**0.010**	-11.43 (-19.07 to -3.80)	-2.94	**0.003**	-14.00 (-25.10 to -2.91)	-2.47	**0.013**

CI = Confidence interval; AD = antidepressants; MS = mood stabilisers

## Discussion

The present study found that AP polypharmacy is associated with QTc interval, and this effect is mediated by AP dose. This finding is noteworthy as clinicians may not be fully aware that AP polypharmacy is associated with high doses, erroneously arguing that AP polypharmacy reduces the total amount of AP medication [18]. The risk of QTc lengthening might therefore be underestimated in patients exposed to two or more concurrent AP drugs.

Risk associated with haloperidol and individual second-generation AP drugs provided additional clinically interesting insights. First, we failed to detect an increased risk associated with haloperidol. We argue that this does not suggest a safe profile of haloperidol but, rather, compliance with current safety warnings [19], which may have led to the selection of individuals without ECG abnormalities. Second, aripiprazole resulted, in comparison with all other AP drugs, associated with a reduced risk of QTc prolongation. This finding is consistent with the conclusions of a systematic review that investigated the cardiac safety of aripiprazole treatment in patients at high risk for torsade [20]. Based on more than 100 preclinical, clinical, and epidemiological studies, the review found strong evidence in QTc data supporting a safe cardiac profile [20]. In addition, experimental data from placebo and head-to-head comparisons between different AP drugs found that aripiprazole was not associated with significant QTc prolongation compared with placebo, and that it was the second best choice in terms of risk of ECG abnormalities second only to lurasidone [21].

These findings should be interpreted bearing in mind some study limitations. A first concern refers to the possibility of confounding, as there are several risk factors for QTc prolongation [2;5]. In this analysis, instead of statistically controlling for some of these risk factors, we restricted the analysis to patients without some characteristics that are known to be associated with QTc lengthening, including cardiovascular disorders and a number of medications. Although this has indubitably increased homogeneity of the study sample, we acknowledge that we were not able to take into consideration other potential contributing factors, such as obesity, malnutrition or hepatic dysfunctions. For example, recent data have shown that AP drugs may be associated with non-alcoholic fatty liver disease (NAFLD) [22], and NAFLD has been shown to increase the risk of a wide spectrum of cardiovascular and cardiac abnormalities, including increased QTc interval [23]. Therefore, AP drugs, in addition to direct cardiac toxicity, may be conducive to increased QTc interval via dysmetabolism and NAFLD. Another limitation relates to the small numbers of patients taking some individual drugs, resulting in both limited statistical power to detect associations with QTc interval and unfeasibility to test associations between QTc prolongation and some “classical” AP combinations (e.g. those involving aripiprazole or clozapine) recommended by evidence-based guidelines [24]. Last, the choice of using ECG data collected for clinical purposes, which may have introduced some heterogeneity in terms of different centres measuring the QT interval in slightly different ways, was motivated by an attempt to resemble as much as possible clinical practice.

Given the high prevalence of AP polypharmacy in real-world clinical practice, still in absence of solid evidence, this study has several implications for routine treatment programmes. Clinicians should bear in mind that since AP polypharmacy is not associated with reduced cumulative dose of AP drugs [25], but the combined equivalent is significantly higher in those receiving AP polypharmacy compared with those on monotherapy, this may actually worsen the QTc interval. Guidelines for the use of AP polypharmacy are progressively moving from recommending it only in patients with clozapine-refractory psychosis [26] towards a more extensive use [27]. Thus, clinicians should consider not only the myriad risk factors for QTc prolongation in their patients, but also that adding a second AP drug may further increase risk as compared with monotherapy. In patients exposed to AP polypharmacy, it may be prudent to monitor QTc before treatment, and then regularly during treatment, depending on the estimated risk.

Clinicians should also consider that AP polypharmacy is not unalterable. Switching from polypharmacy to monotherapy has been shown to be feasible in a majority of patients with schizophrenia, and assertive educational interventions, rather than educational approaches alone, were found to be effective supportive strategies in reducing AP polypharmacy [28].

In terms of implications for research, these data suggest that controlled, high-quality AP combination trials are necessary to determine the effectiveness, safety, and role of AP polypharmacy in the management of severely ill patients. Among safety outcomes, studies should always include measures of cardiac functioning, including the QTc interval.
